# Brain fog of post-COVID-19 condition and Chronic Fatigue Syndrome, same medical disorder?

**DOI:** 10.1186/s12967-022-03764-2

**Published:** 2022-12-06

**Authors:** N. Azcue, J. C. Gómez-Esteban, M. Acera, B. Tijero, T. Fernandez, N. Ayo-Mentxakatorre, T. Pérez-Concha, A. Murueta-Goyena, J. V. Lafuente, Á. Prada, A. López de Munain, G. Ruiz-Irastorza, L. Ribacoba, I. Gabilondo, R. Del Pino

**Affiliations:** 1 Neurodegenerative Diseases Group, Biocruces Bizkaia Health Research Institute, Barakaldo, Spain; 2 Department of Neurology, Cruces University Hospital, Barakaldo, Spain; 3 Department of Neurosciences, University of the Basque Country UPV/EHU, Leioa, Spain; 4 Department of Immunology, Donostia University Hospital, San Sebastián, Spain; 5 Department of Neurology, Donostia University Hospital, San Sebastián, Spain; 6 Department of Neurosciences, Biodonostia Health Research Institute, San Sebastián, Spain; 7 Autoimmune Diseases Research Unit, Biocruces Bizkaia Health Research Institute, Barakaldo, Spain; 8 Department of Internal Medicine, Cruces University Hospital, Barakaldo, Spain; 9 The Basque Foundation for Science, IKERBASQUE, Bilbao, Spain; 10 Spanish Network for the Research in Multiple Sclerosis, Donostia/San Sebastian, Spain

**Keywords:** Brain fog, Cognition, Hyposmia, Myalgic Encephalomyelitis/Chronic Fatigue Syndrome, Neuropsychological impairment, SARS-CoV-2, Post-COVID-19 condition

## Abstract

**Background:**

Myalgic Encephalomyelitis/Chronic Fatigue Syndrome (ME/CFS) is characterized by persistent physical and mental fatigue. The post-COVID-19 condition patients refer physical fatigue and cognitive impairment sequelae. Given the similarity between both conditions, could it be the same pathology with a different precipitating factor?

**Objective:**

To describe the cognitive impairment, neuropsychiatric symptoms, and general symptomatology in both groups, to find out if it is the same pathology. As well as verify if the affectation of smell is related to cognitive deterioration in patients with post-COVID-19 condition.

**Methods:**

The sample included 42 ME/CFS and 73 post-COVID-19 condition patients. Fatigue, sleep quality, anxiety and depressive symptoms, the frequency and severity of different symptoms, olfactory function and a wide range of cognitive domains were evaluated.

**Results:**

Both syndromes are characterized by excessive physical fatigue, sleep problems and myalgia. Sustained attention and processing speed were impaired in 83.3% and 52.4% of ME/CFS patients while in post-COVID-19 condition were impaired in 56.2% and 41.4% of patients, respectively. Statistically significant differences were found in sustained attention and visuospatial ability, being the ME/CFS group who presented the worst performance. Physical problems and mood issues were the main variables correlating with cognitive performance in post-COVID-19 patients, while in ME/CFS it was anxiety symptoms and physical fatigue.

**Conclusions:**

The symptomatology and cognitive patterns were similar in both groups, with greater impairment in ME/CFS. This disease is characterized by greater physical and neuropsychiatric problems compared to post-COVID-19 condition. Likewise, we also propose the relevance of prolonged hyposmia as a possible marker of cognitive deterioration in patients with post-COVID-19.

## Background

Myalgic Encephalomyelitis or Chronic Fatigue Syndrome (ME/CFS) is a complex disorder characterized by physical exertion intolerance, fatigue, cognitive problems, and symptoms derived from autonomic involvement [1], among others. The diagnosis of ME/CFS is based on clinical criteria, those proposed by Fukuda et al. [2] being the most widely used. The cardinal criteria imply a reduction of at least 50% in the physical/cognitive activity compared to the baseline state [2, 3].

The International Classification of Diseases (ICD-11) includes this syndrome within the neurologically-based diseases sections (8E49). Unfortunately, we still lack specific biomarkers or complementary tests beyond ergometry to support this diagnosis. There is no consensus regarding the etiology and pathogenesis of this condition, although the relationship with the immune system, particularly with humoral autoimmunity, is gaining acceptance. The most common precipitating factors in this pathology are infectious diseases, stressful life events or exposure to toxins [4]. The prevalence of ME/CFS is unknown, although a 0.5–2.6% is established [5].

Patients with ME/CFS often experience memory, attention deficits, divided attention, word finding and reasoning difficulties [6]. The most prevalent cognitive deficits seem to be lower processing speed, worse verbal attention [7], and lower sustained attention [8]. Some studies suggest that these deficits are present in around 40–50% of these patients [6]. This inability to concentrate and thought slowness has been defined as ‘brain fog’, being described as “thinking/focusing difficulty” [9]. These symptoms may be due to different factors such as immune abnormalities [10], the presence of autoantibodies [11], orthostatism [12], the breakdown of the blood–brain barrier [13] or neuroinflammation [12]. In addition, they usually present psychopathological symptoms [10, 14], independently of cognitive performance [10, 15], among which depressive and anxious symptoms stand out [10, 14, 15].

Recently, the SARS-CoV-2 infection, known as COVID-19, cause persistent symptoms after the acute phase, being fatigue one of the symptoms that persist the longest [16]. Patients report notable physical and mental fatigue, as well as ‘brain fog’. Muscle pain and weakness, headache, sleep disturbances and palpitations are also common [14, 17]. Different terms were used to refer to this pathology, being it currently named post-COVID-19 condition (U09.9, ICD-10) [18]. This pathology is characterized by fatigue, muscle weakness and pain, dyspnea, chest pain, low-grade fever, cognitive problems, headaches, sleep problems and anxiety [19, 20] which must be present 3 months after infection and last for at least 2 months [18].

Taking into account the extensive evidence of the development of postinfectious ME/CFS after infections like Epstein-Barr virus, cytomegalovirus or Borrelia Burgdorferi, it is expected to find an increase in its incidence in survivors of SARS-CoV-2 [20]. In fact, more than 60% of survivors of SARS-CoV-2 have persistent symptoms [21], being fatigue and dyspnea the most prevalent ones [17]. The prevalence of post-COVID-19 condition is around 14% of those infected [19, 22]. These long-lasting symptoms were significantly lower in vaccinated people, in a range of 11.4% in non-vaccinated people to 5.2% in those who had at least one dose [23].

The objective of this study is to analyze the differences and similarities between CFS/ME and post-COVID-19 condition, focusing especially on the neuropsychological characteristics of both.

## Materials and methods

### Demographics and clinical data

Patients aged 18–85 years with a sufficient understanding and communication skills, with post-COVID-19 condition and with ME/CFS were recruited from those attending the Neurology Department at Cruces University Hospital. Pregnancy and/or lactation, severe trauma, alcoholism, drug addiction, severe heart disease and/or radiological diagnosis of brain structural pathology (tumors, cysts, and malformations) were considered exclusion criteria.

Patients diagnosed with post-COVID-19 condition met the criteria proposed by the NICE guidelines, in which signs and symptoms that develop during or after the infection consistent with COVID-19 continued for more than 12 weeks and were not explained by an alternative diagnosis [24]. For the diagnosis of acute COVID-19, the valid diagnostic methods were a positive nasal PCR, the detection of IgG and/or IgM antibodies against SARS-CoV-2 or a medical report supporting the diagnosis, especially for those patients of the first Spanish wave (from the beginning of the pandemic until June 2020, Spain) who did not undergo diagnostic microbiological tests.

Of the subjects diagnosed with post-COVID-19 condition, 42 patients were infected in the first Spanish wave (between the start of the pandemic and the end of June 2020) in which the predominant variant was SEC8. On the other hand, 20 participants were infected in the second Spanish wave and 8 in the third (between July-December 2020 and from December 2020 to March 2021, respectively). In both, the second and third waves, the predominant variant in Spain was 20E (EU1). The remaining sample was infected in the fourth wave (mid-March to the end of June 2021). The Alpha variant was predominant on those dates.

For patients to be diagnosed with post-COVID-19 condition, any of the following symptoms had to be present at least 3 months after the infection and persisting for at least 2 months [18]: physical fatigue, mental fatigue, palpitations, sensory symptoms and/or dysautonomic symptoms. The exclusion criteria in this group were respiratory disease lasting 12 weeks after the infection, having been admitted to an intensive care unit and/or having had severe bilateral pneumonia or other severe disease manifestations requiring hospitalization. Patients with ME/CFS should have been previously diagnosed by a professional, or meet the criteria mentioned [2]. Those patients with concomitant diseases that could influence the results, as well as those who had previously received some immunomodulatory treatment, were excluded. Regarding the vaccination of these participants, only two patients with a post-Covid-19 condition were previously vaccinated. The vaccinations of these two participants were 2 and 15 days before infection.

For the diagnosis of CFS/ME, we used the criteria proposed by Fukuda et al. [2], in which disabling chronic fatigue (persistent or intermittent) is present for at least 6 months and not explained by any alternative cause, was the main and necessary symptom. In addition, at least four secondary symptoms (odynophagia, myalgias, polyaltralgias, sleep disorder, concentration and memory deficits, lymphadenopathy, headaches, and post-exertional malaise lasting more than 24 h) had to be present.

The study protocol was approved by the Basque Research Ethics Committee [*Comité de Ética de la Investigación con medicamentos de Euskadi* (CEIm-E) (PI2020210)]. All participants gave written informed consent prior to their participation in the study, in accordance with the tenets of the Declaration of Helsinki.

### Neuropsychologic and neuropsychiatric assessment

Neuropsychologic and neuropsychiatric evaluation was performed by an experienced neuropsychologist and neurologist team. Age, sex, years of education and clinically significant variables were recorded for all participants. Overall cognition screening was performed with the Montreal Cognitive Assessment (MoCA). A complete neuropsychological evaluation was carried out to assess the following cognitive domains: attentional verbal and working memory (Digits from Wechsler Adult Intelligence Scale IV [WAIS-IV]), visual attention (Trail Making Test A [TMT A]), sustained attention (Touluose-Piéron Revised [TP-R]), alternating attention (Trail Making Test B [TMT B]) verbal fluency (animals and P), processing speed (Symbol Digit Modality Test [SDMT]) and Salthouse Perceptual Comparison Test [SPCT], cognitive flexibility (Modified Wisconsin Card Sorting Test [M-WCST]), verbal memory (Hopkins Verbal Learning Test- Revised [HVLT-R], visual memory (Brief Visuospatial Memory Test-Revised [BVMT-R]), visuoconstructive capacity (Taylor Complex Figure Test [TCF]), visual perception (Benton Judgment Of Line Orientation [JLO], inhibitory capacity (Stroop Test) and abstraction (similarities from WAIS-IV).

Neuropsychiatric and clinical status were assessed with questionnaires measuring general health (The 36-Item Short Form Health Survey [SF-36]), impact of fatigue (Modified Fatigue Impact Scale [MFIS]), depressive symptoms (the Short Form of Geriatric Depression Scale [GDS]), anxiety symptoms (State-Trait Anxiety Inventory [STAI]), suicidal ideation (Columbia Suicide Severity Rating Scale [C-SSRS]), functional impairment (Karnofsky Performance Status Scale [KPS]), sleep quality (Pittsburgh Sleep Quality Index [PSQI]), the frequency and severity of symptoms (*DePaul* Symptom Questionnaire [DSQ]) and olfactory function with the Brief Smell Identification Test (BSIT).

The neuropsychological and neuropsychiatric evaluation was performed in a single day, lasting approximately one hour and a half. To avoid cognitive fatigue, the DSQ was completed by the patients at home.

### Statistical analysis

Statistical analyses were carried out using IBM SPSS Statistics for Windows, version 23.0 (IBM SPSS, Armonk, NY, USA).

The assumption of normality was analyzed for each of the groups in all the normalized results of the neuropsychological variables using the Shapiro–Wilk test. Group differences in demographical and clinical variables were analyzed with Student’s *t*-test or U Mann–Whitney tests, depending on the fulfillment of the assumption of normality.

We analyzed the differences between groups in the DSQ questionnaire with a Chi square test that allows comparing the distribution of the results in a qualitative variable. The explanatory figure of the DSQ was made using the means of the frequency and severity variables, to see the symptom pattern in each pathology.

Regarding the neuropsychological results, the data were transformed to scalar scores (SS) or to typified scores provided by the test manual. A descriptive analysis and frequencies were performed for the SS of the cognitive tests. We set a cut-off point for cognitive tests, and thus for cognitive impairment, at a scalar score of six. A SS less than six means being more than 1.67 standard deviation below the mean compared to people of the same age and education.

For the cognitive variables’ raw data that fulfilled the normality assumption, the Student's t test was carried out to compare the means between groups. In the case of the tests that did not meet this assumption, the U Mann–Whitney test was performed. The raw data of the cognitive test was also transformed into Z scores for each group to create cognitive composites. These cognitive composites were created as followed: general cognition was composed with the MoCA; verbal fluency by the verbal fluency of animals and words with P; processing speed was composed of the SPCT and SDMT tests; attention domain was made up of the total hits on the TP-R, the Global Index of Attention and Perception (GIAP) of the TP-R, direct digits and the TMT A; verbal memory with the HVLT-R; visual memory by the BVMT-R and the memory of TCF; visuoconstructive ability was composed with the copy of TCF and visuospatial ability with the Benton JLO; and finally, the executive functions were composed with TMT-B, indirect digits (WAIS-IV), the Word-Color subtest of the Stroop test and the M-WCST. We also transformed the neuropsychiatric assessment's results into Z-scores to compare both groups. For these comparisons we used Student’s t-test.

A comparison of means was also made between those ‘probably infected with the SEC8 variant’ (first wave) and with ‘probably infected with the 20E (EU1) variant’ (second and third wave) for neuropsychiatric and cognitive variables, transforming raw scores into Z scores for neuropsychiatric and cognitive variables and comparing them with Student’s t-test. Patients from the fourth wave (possible Alpha variant) were not included due to the scarcity of the sample.

For the BSIT in the post-COVID-19 condition group, we divided the results into three groups following the BSIT normative data, taking into account performance, sex, and age: normal, relatively abnormal, and abnormal. We convert the raw data into Z-scores and create the same cognitive composites only for the post-COVID group, comparing the scores between BSIT groups with the Student's t-test.

In addition, correlations between neuropsychological variables and cognitive domains were analyzed. We also analyzed the correlations between the neuropsychiatric variables along with the disease duration. We used Spearman Rho due to the lack of normality of the tests. Statistical significance was set at *p* < 0.05 (two-tailed). Finally, a stepwise linear regression was carried out to analyze the percentage of variance explained with sleep, fatigue, anxiety-depressive symptoms, suicidal ideation, general health, disease duration, age, education and olfactory function (only for post-COVID-19 patients) on the cognitive variables.

## Results

### Demographic and clinical data

The demographic and clinical data are shown in Table 1. No significant differences were found in age. There were statistically significant differences in education level (U = 1191.00; *p* = 0.046) with the post-covid group having more years of formal education. Statistically significant differences were also found in the proportion of women between groups (*χ*^2^ = 8.29; *p* = 0.004), 92.9% of ME/CFS and 69.9% of post-COVID-19 patients were women. Most of the patients were Caucasian, being only one patient in the post-COVID-19 group an another in ME/CFS who did not identify with any of the U.S. Office of Management and Budget’s race categories. These differences are not expected to have an impact on the results, as these were corrected for age, education and sex.

**Table 1:** Demographic and clinical features and neuropsychological assessment

Demographic and clinical characteristics	ME/CFS (n = 42) RS M (SD)	Post-COVID-19 condition (n = 73) RS M (SD)	Statistics
Age	43.50 (8.24)	44.36 (9.47)	U = 1397.50
Women, *n* (%)	39 (92.9)	51 (69.9)	*χ*^2^ = 8.29**
Caucasian race,* n* (%)	41 (97.6)	72 (98.6)	*χ*^2^ = .159
Education, years	14.78 (4.31)	16.34 (3.33)	U = 1191.00*
Disease duration, months	85.57 (100.71)	12.39 (5.55)	U = 2592.50**
Neuropsychologic assessment			
General cognition			
MoCA	25.52 (2.95)	25.09 (3.06)	U = 1648.50
Verbal fluency			
Animals	18.95 (4.96)	19.89 (5.98)	t = .86
P	14.16 (4.71)	13.84 (4.80)	U = 1603.50
Visual processing speed			
SDMT	42.73 (11.47)	47.23 (10.93)	U = 1149.50*
SPCT 3	14.30 (4.25)	16.75 (5.26)	U = 1073.50**
SPCT 6	8.67 (2.85)	8.91 (3.12)	U = 1434.50
Attention			
Verbal attention			
Direct digits (WAIS-IV)	5.64 (1.10)	5.56 (1.06)	U = 1555.00
Visual attention			
TMT-A	40.78 (16.75)	38.41 (14.50)	U = 1674.5
Sustained attention (TP-R)			
Hits	152.57 (51.54)	179.78 (52.38)	t = 2.69**
Omissions	43.00 (41.73)	36.82 (28.20)	U = 1563.00
Mistakes	5.33 (15.17)	0.89 (2.37)	U = 1677.00
GIAP	106.30 (64.84)	141.95 (58.49)	U = 999.80**
ICI	96.00 (14.81)	98.55 (5.27)	U = 1492.00
Visuoconstructive abilities			
TCF copy	32.64 (3.12)	32.05 (3.17)	U = 1728.00
Visuospatial perception			
Benton JLO	22.57 (4.32)	24.52 (4.98)	U = 1064.5**
Visual memory			
TCF memory	20.39 (6.30)	21.73 (5.53)	t = 1.19
Trial 1 BVMT-R	4.73 (2.29)	5.63 (3.54)	t = 1.46
Trial 1–3 BVMT-R	20.90 (6.46)	22.38 (7.37)	U = 1334.00
Trial 4 BVMT-R	8.35 (3.31)	8.31 (2.70)	U = 1507.50
DI BVMT-R	5.66 (.78)	5.64 (1.04)	U = 1505.50
Verbal memory (HVLT-R)			
Trial 1	5.35 (1.62)	5.31 (1.55)	U = 1530.50
Total (1–3)	23.23 (4.50)	22.57 (5.92)	t = -.63
Trial 4	8.28 (2.47)	7.90 (2.83)	U = 1608.00
DI	10.02 (1.63)	9.53 (2.47)	U = 1655.50
Executive functions			
Abstraction			
Similarities (WAIS-IV)	20.33 (4.30)	22.05 (5.65)	U = 1298.50
Alternating attention			
TMT-B	99.61 (49.61)	86.65 (42.68)	U = 1877.00*
Working memory			
Indirect digits (WAIS-IV)	4.07 (.89)	4.39 (1.69)	U = 1262.50
Visual processing speed and inhibition (Stroop Test)			
Word	88.38 (19.96)	93.49 (24.97)	U = 1322.50
Color	61.52 (13.12)	66.32 (13.79)	t = 1.83
Word-color	36.67 (10.58)	40.63 (10.249	t = 1.97
Cognitive flexibility (M-WCST)			
Categories	5.78 (1.40)	6.12 (1.35)	U = 1288.00
Perseverative mistakes	1.90 (2.34)	1.76 (2.28)	U = 1607.00
Total mistakes	7.64 (5.08)	7.13 (5.71)	U = 1680.50

The Student’s t and *U* Mann–Whitney scores were obtained using the SS, except in those cases in which only RS was available

*Benton JLO* Benton Judgment Line Orientation; *BVMT-R* Brief Visuospatial Memory Test-Revised; *HVLT-R* Hopkins Verbal Learning Test-Revised; *GIAP* Global Index of Attention and Perception; *ICI* Impulsivity Control Index; *M* Mean; *MocA* Montreal Cognitive Assessment; *M-WCST* Modified Wisconsin Card Sorting Test; *RS* raw score; *SD* standard deviation; *SDMT* Symbol Digit Modality Test; *SPCT* Salthouse Perception Comparison Test; *TCF* Taylor Complex Figure; *TMT* Trail Making Test; *TP-R* Toulouse Piéron-Revised Test*; WAIS IV* Wechsler Adult Intelligence Scale IV

^*^p < 0.05 **p < 0.01 ***p ≤ 0.001

### Symptomatology

**Fig. 1:**
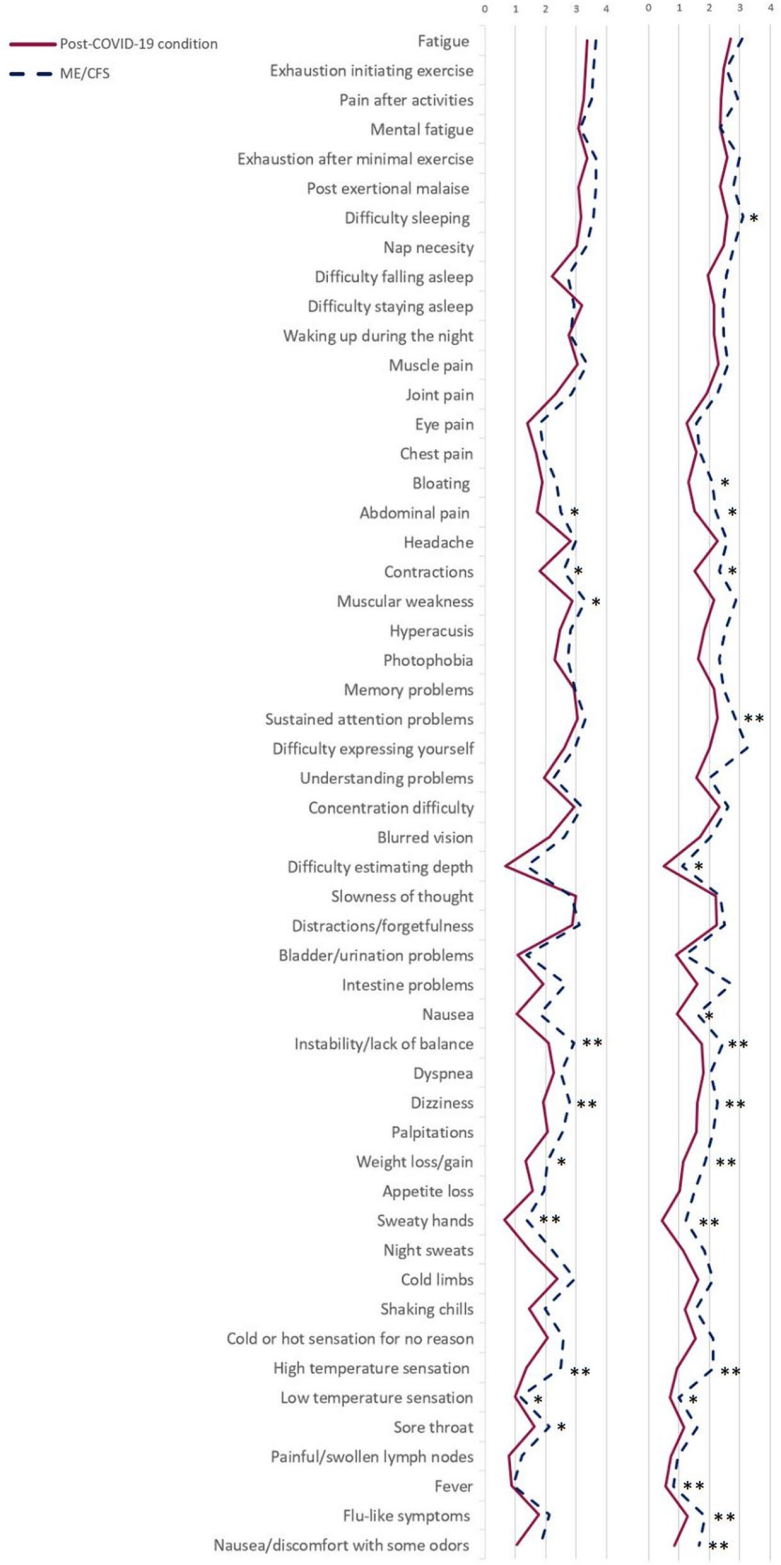
Severity and frequency of symptoms (DSQ). *p < 0.05 **p ≤ 0.01; 0: Lack of symptoms; 1: Mild; 2: Moderate; 3: Severe; 4: Very severe; F: Frequency; ME/CFS: Myalgic Encephalomyelitis/Chronic Fatigue Syndrome; S: Severity

Initially, we analyzed the most prevalent symptoms in both conditions. Figure 1 shows the frequency and severity of the symptoms evaluated in the DSQ with a comparison between both groups. Fatigue, the feeling of heaviness or exhaustion when exercising, post exertional malaise, difficulty sleeping and mental fatigue were the most prevalent symptoms. Significant differences between the frequency and severity showed a more severe disease course in the ME/CFS group (Fig. 1). These significant differences were found in the frequency of abdominal pain (*χ*^2^ = 11.39; *p* = 0.022), contractions (*χ*^2^ = 10.14; *p* = 0.038), instability/lack of balance (*χ*^2^ = 14.30; *p* = 0.006), dizziness or fainting (*χ*^2^ = 13.53; *p* = 0.009), unintentional weight loss/gain (*χ*^2^ = 12.91; *p* = 0.012), sweaty hands (*χ*^2^ = 18.51; *p* = 0.001), hot sensation (*χ*^2^ = 18.47; *p* = 0.001), cold sensation (*χ*^2^ = 10.11; *p* = 0.039) and sore throat (*χ*^2^ = 9.54; *p* = 0.049). Significant differences were also found in the severity of some of the symptoms evaluated, including the feeling of unrefreshing sleep (*χ*^2^ = 9.85; *p* = 0.043), bloating (*χ*^2^ = 10.65; *p* = 0.031), abdominal pain (*χ*^2^ = 10.93; *p* = 0.027), contractions (*χ*^2^ = 12.87; *p* = 0.012), muscle weakness (*χ*^2^ = 10.6; *p* = 0.039), the ability to maintain attention (*χ*^2^ = 15.13; *p* = 0.004), problems with depth perception (*χ*^2^ = 10.03; *p* = 0.040), nausea (*χ*^2^ = 11.72; *p* = 0.020), instability/lack of balance (*χ*^2^ = 16.11; *p* = 0.003), dizziness or fainting (*χ*^2^ = 14.62; *p* = 0.006), weight loss/gain (*χ*^2^ = 13.32; *p* = 0.010), sweaty hands (*χ*^2^ = 18.12; *p* = 0.001), feeling of having a high temperature (*χ*^2^ = 26.91; *p* = 0.000) or low temperature (*χ*^2^ = 9.78; *p* = 0.044), flu-like symptoms (*χ*^2^ = 15.30; *p* = 0.004) and discomfort/nausea from certain odors (*χ*^2^ = 11.25; *p* = 0.024).

### Neuropsychological impairment

**Fig. 2:**
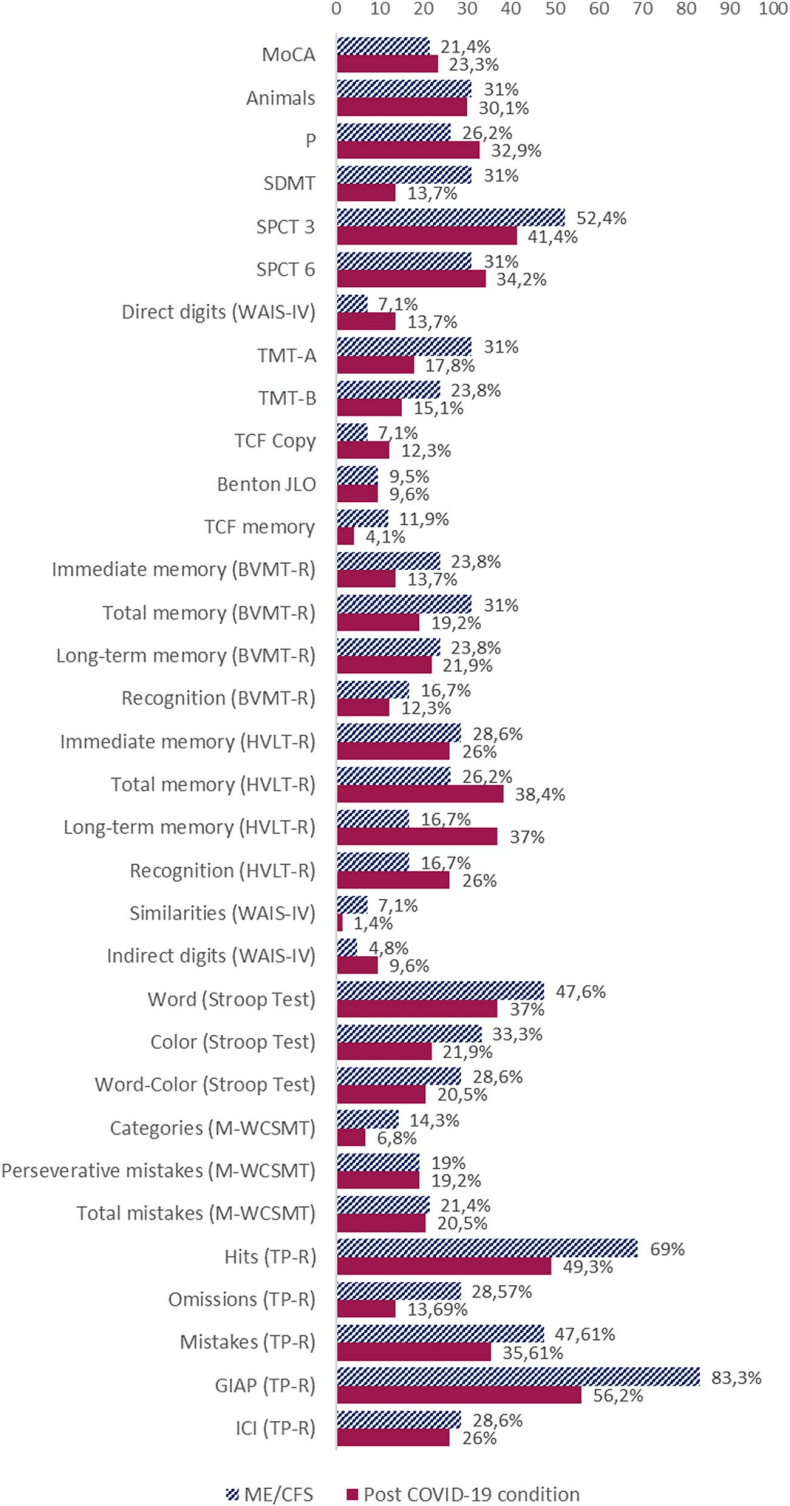
Percentage of patients with SS less than six in the cognitive tests and with deficient TP-R test results. Benton JLO: Benton Judgment Line Orientation test; BVMT-R: Brief Visuospatial Memory Test-Revised; GIAP: Global Index of Attention and Perception; HVLT-R: Hopkins Verbal Learning Test-Revised; ICI: Impulsivity Control Index; MoCA: Montreal Cognitive Assessment; M-WCST: Modified Wisconsin Card Sorting Test; SDMT: Symbol Digit Modality Test; SPCT: Salthouse Perception Comparison Test; TCF: Taylor Complex Figure; TMT: Trail Making Test; TP-R: Toulouse-Piéron-Revised test; WAIS-IV: Wechsler Adult Intelligence Scale IV

Regarding the cognitive performance of the groups, statistical analysis of cognitive outcomes revealed that the most predominant affected cognitive domain was the sustained attention capacity (Fig. 2), which was detected in 56.2% of patients with post-COVID-19 condition vs. 83.3% of patients with ME/CFS, with statistically significant differences between both groups in the TP-R test (Table 1), being ME/CFS patients the most affected ones. These deficits were followed by processing speed, memory and ability to learn verbal material.

**Fig. 3:**
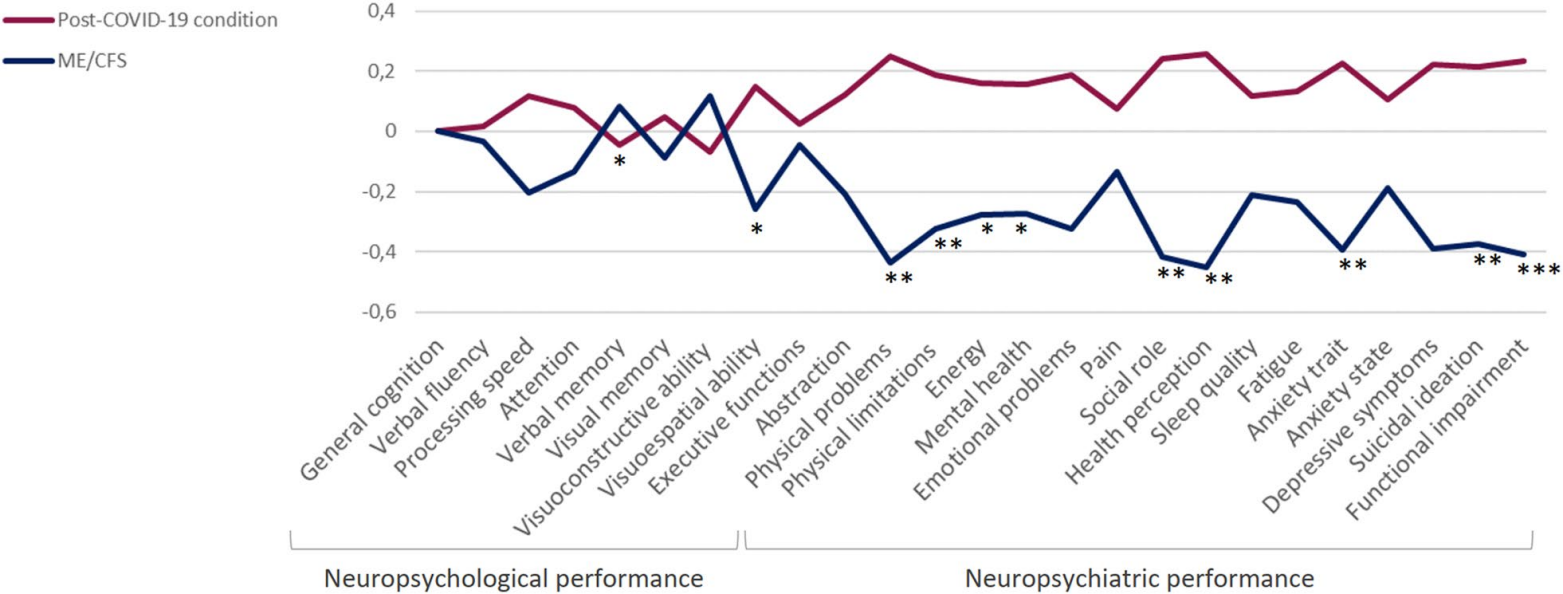
Z scores by cognitive domains and neuropsychiatric assessment. *p < 0.05; **p < 0.01, *** *p* < 0.001; C-SSRS: Columbia Suicide Severity Rating Scale; GDS: Geriatric Depression Scale; ME/CFS: Myalgic Encephalomyelitis/Chronic Fatigue Syndrome; MFIS: Modified Fatigue Impact Scale; PSQI: Pittsburgh Sleep Quality Index; SF-36: The 36-Item Short Form Health Survey; STAI: State-Trait Anxiety Inventory

The differences between groups in cognitive domains were analyzed using Z-scores (Fig. 3). The scores of the neuropsychological and neuropsychiatric evaluations imply a worse cognitive performance the lower the Z. There were statistically significant differences in attention (t = 2.12; *p* = 0.037) and visuospatial ability (t = 2.12; *p* = 0.037) with lower scores in the ME/CFS group. The ME/CFS group performed worse in most cognitive domains, except for visuoconstructive ability and verbal memory.

### Neuropsychiatric performance

The Z-scores analyzed for neuropsychiatric variables show significantly worse mental health in ME/CFS patients (Fig. 3). SF-36 questionnaire showed that ME/CFS patients perceived more physical limitations (t = 2.70; *p* = 0.008), lower energy (t = 2.29; *p* = 0.000), poorer mental health (t = 2.27; *p* = 0.025), more impact of health on their social interactions (t = 3.57; *p* = 0.001), and a worse general health perception (t = 3.87; *p* = 0.000). ME/CFS patients had also greater levels of anxiety trait (t = -3.35; *p* = 0.001), more depressive symptoms (t = -3.28; *p* = 0.001), and a greater suicidal ideation (t = − 3.17; *p* = 0.002) compared to the post-COVID-19 patients (Table 2).

**Table 2:** Neuropsychiatric assessment

Neuropsychiatric assessment	ME/CFS (n = 42) RS M (SD)	Post-COVID-19 condition (n = 73) RS M (SD)	Statistics
Health perception (SF-36)			
Physical functioning	32.14 (21.24)	49.21 (24.71)	t = 3.74***
Limitations due to physical problems	9.22 (14.16)	22.22 (29.23)	U = 2077.50*
Pain	27.67 (30.18)	33.28 (24.64)	U = 1248.00
Social role	13.27 (22.27)	32.46 (30.45)	U = 881.00***
Mental health	49.64 (24.99)	60.63 (25.04)	U = 1141.00*
Limitations due to emotional problems	61.50 (33.58)	73.13 (29.85)	U = 1220.50
Energy, fatigue	9.37 (12.51)	15.90 (15.81)	U = 1111.50*
Health perception	23.39 (16.34)	38.13 (21.29)	U = 881.50***
Sleep quality			
PSQI	12.85 (5.04)	11.26 (4.64)	U = 1752.50
Fatigue			
MFIS	68.09 (12.74)	62.68 (15.41)	U = 1847.50
Performance status			
Karnofsky scale	68.09 (8.33)	73.97 (8.93)	U = 1025.00***
Depression			
GDS	9.35 (3.21)	6.97 (3.97)	U = 2070.50**
Suicidal ideation			
C-SSRS	0.83 (1.32)	0.24 (0.66)	U = 1906.50**
Anxiety (STAI)			
Anxiety trait	26.97 (17.12)	27.12 (14.12)	U = 1133.50**
Anxiety state	31.42 (15.32)	17.08 (14.04)	t = − 1.52

The Student’s *t* and *U* Mann–Whitney scores were obtained using the SS, except in those cases in which only RS was available

*C-SSRS* Columbia Suicide Severity Rating Scale; *GDS* Geriatric Depression Scale; *M* Mean; *MFIS* Modified Fatigue Impact Scale; *PSQI* Pittsburgh Sleep Quality Index; *SD* standard deviation; *SF-36* The 36-Item Short Form Health Survey; *STAI* State-Trait Anxiety Inventory

^*^p < 0.05 **p < 0.01 ***p ≤ 0.001

Statistically significant differences were only found between the ‘probable SEC8 variant’ and ‘probable 20E (EU1) variant’ groups for the mental health subscale (SF-36), with variant 20E (EU1) being the one that obtained a lower result (t = 2.67; *p* = 0.01), and therefore worse mental health.

### Olfactory, neuropsychological and neuropsychiatric performance

**Fig. 4:**
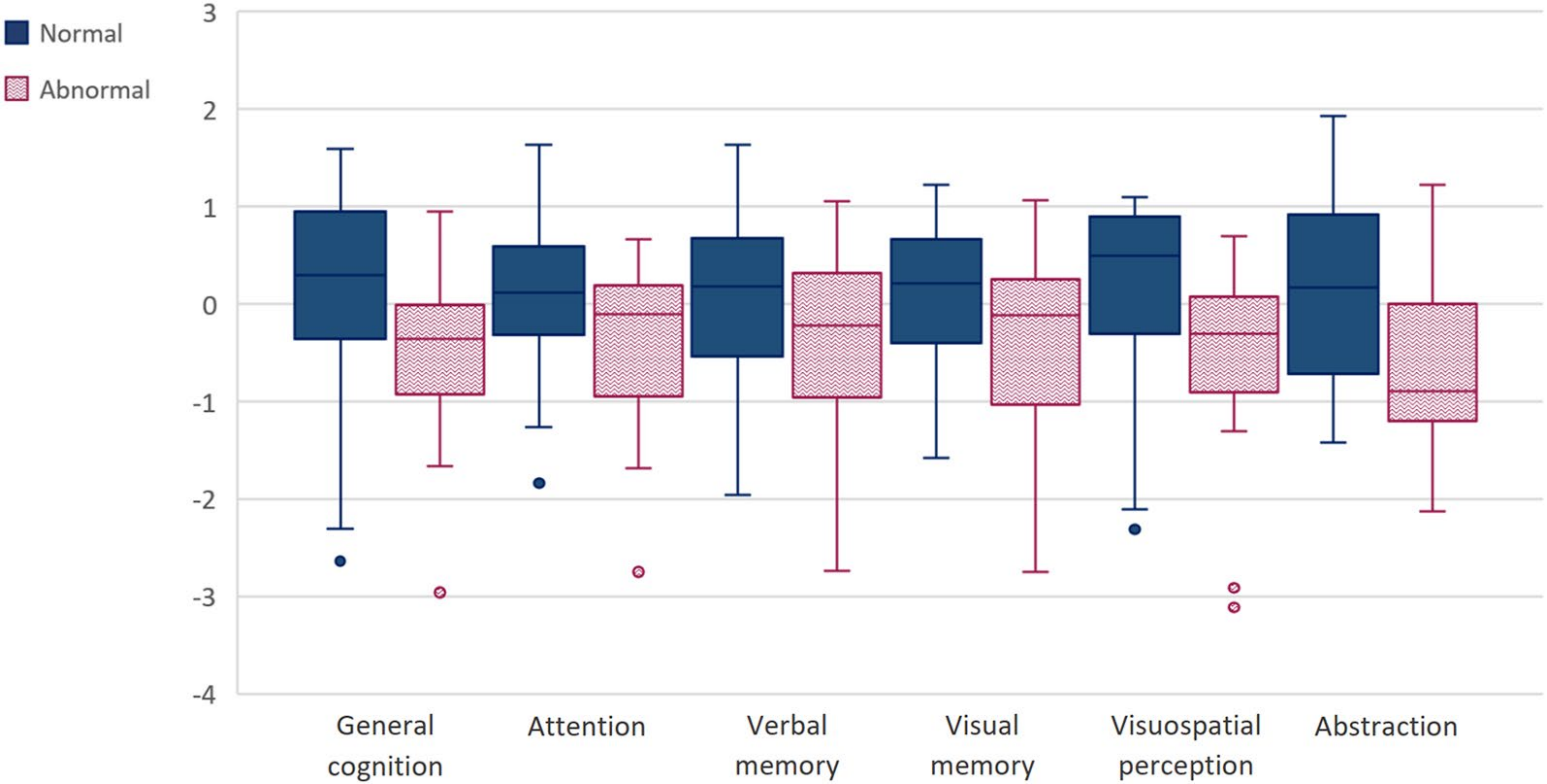
Distribution of cognitive performance according to BSIT results. Benton JLO: Benton Judgement Line Orientation; MoCA: Montreal Cognitive Assessment; SDMT: Symbol Digit Modality Test

We found statistically significant correlations between post-COVID-19 patient’s cognitive performance and BSIT raw data. Taking into account that the ability to smell varies by sex and age, the raw data were corrected according to these two variables, creating three groups of results: normal, relatively abnormal and abnormal, as proposed in the BSIT manual. The ‘normal’ BSIT group was composed of 53 patients, 3 patients were in the ‘relatively abnormal’ group, and 15 in the ‘abnormal’ group. The ‘normal’ group had a mean of 9.96 (SD = 0.99), the ‘relatively abnormal’ group had a mean of 7.67 (SD = 0.58), and the ‘abnormal’ group had a mean of 6.47 (SD = 0.99) on the BSIT. This test’s results correlated with general cognition (MoCA; r = 0.24, *p* = 0.043), visual processing speed (SPCT 3; r = 0.24, *p* = 0.043, and SDMT; r = 0.29, *p* = 0.014), verbal memory (HVLT-R 1–3; r = 0.23; *p* = 0.049), visual memory (BVMT-R discrimination index; r = 0.26; *p* = 0.031), attentional capacity (TMT A; r = -0.39; *p* = 0.001) and visuospatial ability (Benton JLO; r = 0.35; *p* = 0.003). The relatively abnormal group was discarded due to the small sample, as well as the absence of statistically significant differences with the rest of the groups. Statistically significant differences were found between the groups with normal results and those with an abnormal result in general cognition (t = 2.43; *p* = 0.020), attention (t = 2.46; *p* = 0.050), verbal memory (t = 2.14; *p* = 0.036), visual memory (t = 2.29; *p* = 0.025), visuospatial perception (t = 3.04; *p* = 0.014), and abstraction capacity (t = 3.10; *p* = 0.005) (Fig. 4).

We also analyzed correlations between cognitive performance and neuropsychiatric status (Table 3). Table 3 shows correlations between cognitive and neuropsychiatric variables ranged from Rho = 0.30 to Rho = 0.53 (*p* ≤ 0.01). In the post-COVID-19 condition group, sleep quality, fatigue and pain correlated with general cognition, phonological verbal fluency, visual processing speed, long-term verbal memory, attention in the word Stroop subtest and inhibition capacity in the word-color Stroop subtest. Anxiety levels correlated with general cognition, visual processing speed, attention capacity (TMT A), sustained attention and visual recognition. Visual recognition also correlated with the depressive symptoms in GDS. In the ME/CFS patients, anxiety levels were the ones that presented the most statistically significant correlations with the different cognitive domains, more specifically, they seem to influence the capacity for phonological verbal fluency, attention (TMT A), sustained attention and cognitive flexibility (perseverative errors in M-WCST).

**Table 3:**
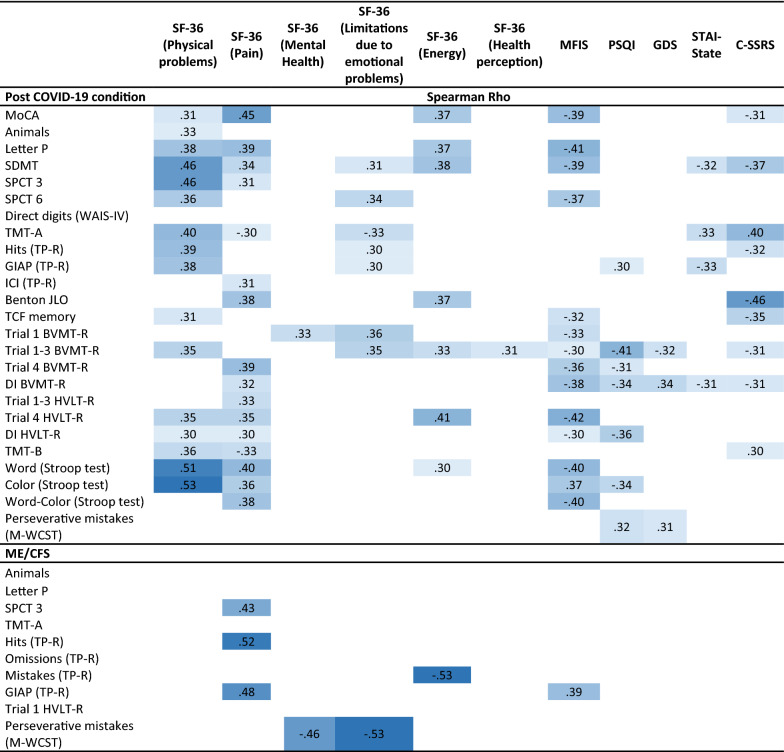
Significant Spearman correlation coefficients between neuropsychiatric and cognitive variables

The values in the table are displayed in different tones, with higher correlations being darker

*BVMT-R* Brief Visuospatial Memory Test-Revised; *C-SSRS* Columbia Suicide Severity Rating Scale; *DI* Discrimination Index; *GDS* Geriatric Depression Scale; *HVLT-R* Hopkins Verbal Learning Test-Revised; *GIAP* Global Index of Attention and Perception; *MFIS* Modified Fatigue Impact Scale; *MoCA* Montreal Cognitive Assessment; *M-WCST* Modified Wisconsin Card Sorting Test; *PSQI* Pittsburgh Sleep Quality Index; *SDMT* Symbol Digit Modality Test; *SF-36* The 36-Item Short Form Health Survey; *SPCT* Salthouse Perception Comparison Test; *STAI* State-Trait Anxiety Inventory; *TCF* Taylor Complex Figure; *TMT* Trail Making Test; *TP-R* Toulouse Piéron-Revised Test; *WAIS* *IV* Wechsler Adult Intelligence Scale IV

All values are *p* ≤ 0.01

Correlations between neuropsychiatric outcomes are shown in Table 4. Data range from Rho = 0.25 to Rho = 0.70. Patients with post-COVID-19 condition presented lower independence and general health (Karnofsky scale) the greater the physical limitations, pain and fatigue. In these patients, the greater the depressive symptomatology, the worse the quality of sleep and the perception of their health. Fatigue levels, in turn, correlated mostly with physical problems. On the other hand, the ME/CFS group showed less independence and general health (Karnofsky scale) the greater the physical limitations, pain and anxiety. The perception of health correlated inversely with anxious and depressive symptomatology. Fatigue levels correlated mainly with anxiety- state. In both groups, mental health (SF-36) correlated with anxiety (STAI-state) and depression (GDS). Pain and physical limitations were also related in both groups. Limitations in maintaining a social life due to health issues were also related to fatigue and physical limitations, likewise, depressive symptomatology and anxiety symptoms were related to suicidal ideation in both groups.

**Table 4:**
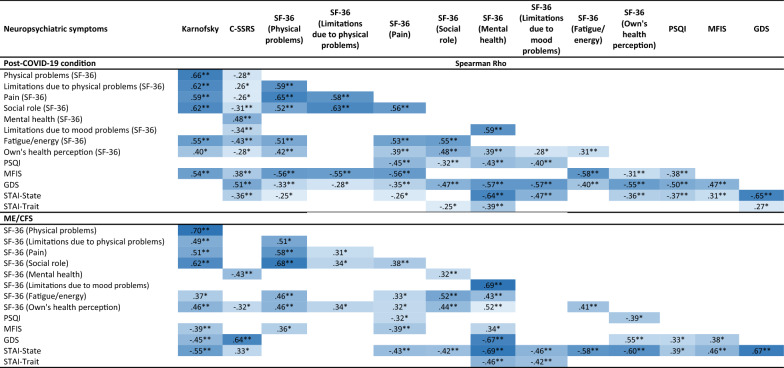
Significant Spearman correlation coefficients between neuropsychiatric variables

The values in the table are displayed in different tones, with higher correlations being darker

*C-SSRS* Columbia Suicide Severity Rating Scale; *GDS* Geriatric Depression Scale; *MFIS* Modified Fatigue Impact Scale; *PSQI* Pittsburgh Sleep Quality Index; *SDMT* Symbol Digit Modality Test; *SF-36* The 36-Item Short Form Health Survey; *SPCT* Salthouse Perception Comparison Test; *STAI* State-Trait Anxiety Inventory

*p < 0.05; **p < 0.01

### Linear regression analysis

**Fig. 5:**
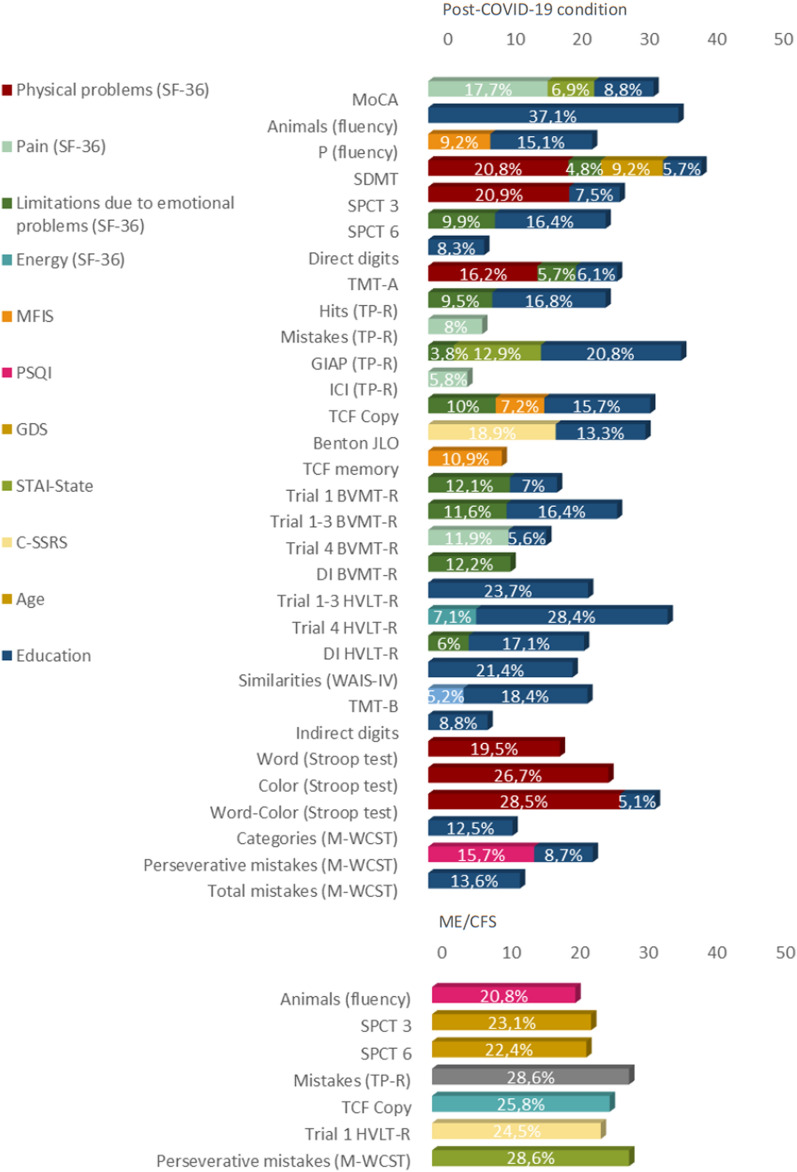
Percentage of variance correlated with cognitive performance. BVMT-R: Brief Visuospatial Memory Test-Revised; DI: discrimination Index; GDS: Geriatric Depression Scale; GIAP: Global Index of Attention and Perception; HVLT-R: Hopkins Verbal Learning Test-Revised; ICI: Impulsivity Control Index; MFIS: Modified Fatigue Impact Scale; MoCA: Montreal Cognitive Assessment; M-WCST: Modified. Wisconsin Card Sorting Test; PSQI: Pittsburgh Sleep Quality Index; SDMT: Symbol Digit Modality Test; SPCT: Salthouse Perceptual Comparison Test; TCF: Taylor Complex Figure; TMT: TP-R: Toulouse-Piéron-Revised Test; WAIS-IV: Weschler Adult Intelligence Scale-IV

Taking into account the correlations between education level, age, disease duration, cognition and neuropsychiatric variables, we made a stepwise linear regression analysis. Education, physical problems, pain, fatigue, sleep quality, depressive symptoms, anxiety and suicidal ideation, largely explained the variance of the cognitive deficits found in both groups, with a range of 3.8–37.1% explained variance (Fig. 5).

Post-COVID-19 condition patients’ education explained the highest percentage of variance in the cognitive performance (from 5.1% to 37.1%). Physical problems explained 28.5% and 16.2% of the variance in attention (Stroop test and TMT-A, respectively) and 20.9% of visual processing speed (SPCT). Pain (SF-36) was the variable that most explained the variance of sustained attention (TP-R errors) (8%), also explaining 5.8% of impulsivity (TP-R Impulsivity Control Index).

On the other hand, visual cognitive tests' performance was explained mainly by the limitation due to emotional problems (SF-36), which explained 12.2% of the variance of visual recognition (BVMT-R Discrimination Index), and fatigue (MFIS), which explained 10.9% of visual memory (TCF 3' memory), 9.2% of phonological verbal fluency (letter P), and 7.2% of visuoconstructive capacity (TCF copy).

Finally, sleep quality (PSQI) explained 15.7% of executive functions (M-WCST perseverative errors), anxiety state (STAI) explained 12.9% of sustained attention (TP-R) and 6.9% of general cognition (MoCA), and suicidal ideation (C-SSRS) explained 18.9% of the visual perception capacity variance (Benton JLO).

Regarding the ME/CFS group, energy/fatigue (SF-36) explained 28.6%. of the variance of sustained attention (TP-R errors). Sleep quality (PSQI) 20.8% of semantic verbal fluency (animals), anxiety levels (STAI-state) 28.6% of executive functions (M-WCST perseverative errors), and suicidal ideation (C-SSRS) 24.5% of immediate verbal memory capacity (HVLT-R trial 1).

## Discussion

Cognitive performance and neuropsychiatric symptoms were analyzed in patients with ME/CFS and post-COVID-19 condition. Although the possibility that patients who had COVID-19 could develop ME/CFS had previously been considered [25], this is the first study, to our knowledge, analyzing and comparing both conditions.

Regarding clinical symptomatology, high physical fatigue, exhaustion when initiating exercise, post-exertional malaise, difficulty sleeping, myalgia, muscle weakness and cold limbs were the most common non-cognitive symptoms in both groups, along with cognitive problems related with lack of concentration, sustained attention problems, mental fatigue, slowness of thought, forgetfulness and frequent distractions [17]. Fatigue, one of the most common and invalidating symptoms in these pathologies, may be due to autonomic nervous system involvement [26, 27]. As fatigue is a subjective variable, there are few studies that analyze its impact on cognition in healthy adults. The effects of exercise-induced fatigue may be task-specific, affecting mostly to perceptual tasks that involve relatively automatic processing [28].

### Cognitive, neuropsychiatric and olfactory impairment

Both conditions presented slow processing speed, deficient sustained attention and verbal memory impairment [29]. Statistically significant differences were found in attention and visual perception, with the ME/CFS group presenting the largest impairment. Therefore, we can understand that the brain fog could have similar cognitive deficits in both groups, being it mainly a reduction in the attentional capacity and a lower processing speed. This mental fatigue or brain fog seems to be closely related to physical fatigue.

Deficits in the attentional capacity may be due to different factors depending on the condition. Our results pointed out that physical fatigue, pain, anxiety, suicidal ideation, sleep quality and fatigue levels were related with cognitive impairment. Specifically, fatigue, physical problems, pain and sleep quality were the most prevalent correlations in the cognitive performance of post-COVID-19 group. In the ME/CFS patients, anxiety, sleep quality, energy levels and suicidal ideation were the most prevalent correlations. In the group of patients with ME/CFS group, anxiety inversely correlated with executive functions (M-WCST perseverative errors) [30, 31]. Energy levels of the SF-36 related to the number of mistakes made in the sustained attention test. Even so, pain did not explain the cognitive performance in ME/CFS group. Some of these patients may suffer from fibromyalgia, in this case, it would be interesting to verify if it is a subgroup of patients with greater cognitive impairment associated with this disease. With regard to the post-COVID-19, those with more physical problems and more pain appeared to perform worse on some cognitive domains. Higher levels of pain were related with poorer long-term visual memory and number of errors in sustained attention test. Chronic pain causes a decrease in attentional capacity and processing speed [30, 31], prolonged pain may also cause a reduction of the gray matter which also could lead to the worsening of the general cognitive performance [32]. In addition, we must add the cognitive deterioration related to the olfactory capacity in post-COVID-19 patients. Other studies have revealed the relationship between prolonged hyposmia after SARS-CoV-2 infection and cognitive impairment [33–35]. According to our results, general cognition, processing speed, abstraction capacity and visuospatial capacity are related to olfactory function in these patients.

Sleep quality also correlated the performance in cognitive flexibility the post-COVID-19 condition group while in the ME/CSF group, it related to the semantic verbal fluency. All these tests require the involvement of the prefrontal lobe for its adequate performance, so that the quality of sleep could be closely related to the prefrontal activity. Previous studies have already shown the implication of sleep quality in adults without cognitive problems [36, 37]. In the group of patients with ME/CFS, poorer sleep quality was related to worse performance on executive function tasks, although it was not related to working memory. Previous studies had already revealed the relationship between poorer sleep quality and performance in this type of task [37].

### Disease course, possible outcomes and future research

Disease duration did not correlate with any of the cognitive tests, nonetheless, some studies associate ME/CFS with small fiber neuropathies, so the absence of progressive cognitive impairment does not imply the absence of neurodegeneration, since the damage of small fibers can progress and thus affect various functions as sudomotor, genitourinary, cardiovascular, pupillary or gastrointestinal functions [38, 39]. The evaluation of damage in the autonomic nervous system and the denervation of small fibers could be useful to discern phenotypes of patients. Some syndromes caused by the alteration of the vegetative nervous system, such as Postural Orthostatic Tachycardia Syndrome (POTS), are common in ME/CFS [9]. This syndrome can be caused by an autoimmune disease or be caused by an infectious disease, as recently seen after SARS-CoV-2 infection [40]. Other studies showed that some of ME/CFS patients notice a progressive worsening over time compared to the majority of patients with this condition, who refer a stable disease path with some fluctuations or relapses [41]. Therefore, it would be convenient to carry out a dysautonomic study that would allow us to better discern the dysautonomic symptoms associated with both syndromes and possible phenotypes in both conditions. In the case of post-COVID-19 condition, we are facing a unique situation for studying post-infectious fatigue since its inception, for this, it would be crucial to carry out a follow-up study in these patients and assess the progression and course of the disease.

Regarding the neuropsychiatric status, ME/CFS patients showed greater anxiety, depressive symptoms, suicidal ideation and a general worse perception of their health. These results along with the cognitive performance showed a more severe disease curse in ME/CFS.

The level of functionality in patients with post-COVID-19 was mainly related to a more physical than a neuropsychiatric sphere, correlating mainly with physical limitations, pain, and physical and mental fatigue. In the case of ME/CFS, the level of functionality was mostly related to physical capacity, pain and anxiety. Therefore, pain reduction and physical recovery should be objective variables in these populations, trying to avoid a sedentary lifestyle that can produce greater physical deconditioning. Both anxious and depressive symptoms should be evaluated and treated in both conditions, since both are closely related to suicidal ideation [42–44].

### Limitations

Although the relevance information of these results, this study presented limitations. We found a disparity of the sample in terms of the percentage of women. The absence of a control group must also be taken into account. Despite this, these limitations were partially corrected by converting the cognitive tests results to normalized scores. The study participants did not have genome sequencing tests to determine the variant they suffered from, so we can only deduce the probability of having been infected by a specific variant taking into account the date of infection. No analysis was performed comparing the results between vaccinated and unvaccinated post-COVID-19 patients due to the small sample of vaccinated participants. Likewise, taking into account the predominance of Caucasians and unvaccinated participants in the study, we cannot know if the symptomatology varies between subjects of other races and vaccinated people. Future studies could clarify these questions. On the other hand, a larger sample of patients with different levels of hyposmia could help discern the cognitive impairment associated with the olfactory function in post-COVID-19 patients.

## Conclusion

In conclusion, we found that both syndromes curse with similar cognitive impairment, being patients with ME/CFS the ones with the worst performance in attention tasks and visual perception. Brain fog in both conditions was characterized by a reduced attention capacity and a slower visual processing speed. Both syndromes also presented high levels of fatigue, poor sleep quality, anxiety, and depressive symptoms, being the physical problems, pain, fatigue levels, anxiety and suicidal ideation the factors that most influence cognitive performance. Physical symptoms as dizziness, instability, high temperature sensation, and muscle weakness were more prevalent in ME/CFS. These results suggested a similar cognitive and symptomatologic pattern in both groups, with punctual differences that characterize each pathology, being ME/CFS, according to our results, a disease characterized by greater physical and neuropsychiatric problems.

## Data Availability

Not applicable.

## Abbreviations

BSIT: Brief Smell Identification Test
BVMT-R: Brief Visuospatial Memory Test-Revised
C-SSRS: Columbia Suicide Severity Rating Scale
DSQ: *DePaul* Symptom Questionnaire
GDS: Geriatric Depression Scale
HVLT-R: Hopkins Verbal Learning Test-Revised
ICD: International Classification of Diseases
JLO: Judgment of Line Orientation
KPS: Karnofsky Performance Status Scale
ME/CFS: Myalgic Encephalomyelitis/Chronic Fatigue Syndrome
MFIS: Modified Fatigue Impact Scale
MoCA: Montreal Cognitive Assessment
M-WCST: Modified Wisconsin Card Sorting Test
PSQI: Pittsburgh Sleep Quality Index
SDMT: Symbol Digit Modality Test
SF-36: The 36-Item Short Form Health Survey
SPCT: Salthouse Perceptual Comparison Test
STAI: State-Trait Anxiety Inventory
TCF: Taylor Complex Figure
TMT: Trail Making Test
TP-R: Touluose-Piéron Revised
WAIS-IV: Wechsler Adult Intelligence Scale IV

## References

[1] Rivera MC, Mastronardi C, Silva-Aldana CT, Arcos-Burgos M, Lidbury BA (2019). Myalgic encephalomyelitis/chronic fatigue syndrome: a comprehensive review. Diagnostics.

[2] Fukuda K, Straus SE, Hickie I, Sharpe MC, Dobbins JG, Komaroff A (1994). The chronic fatigue syndrome: a comprehensive approach to its definition and study. Ann Intern Med.

[3] Carruthers BM, van de Sande MI, de Meirleir KL (2011). Myalgic encephalomyelitis: international consensus criteria. J Intern Med.

[4] Friedberg F, Ph D. ME/CFS . A primer for clinical practitioners members of the IACFS/ME primer writing committee. International association for chronic fatigue. Published online 2012.

[5] Wessely S, Chalder T, Hirsch S, Wallace P, Wright D (1997). The prevalence and morbidity of chronic fatigue and chronic fatigue syndrome: a prospective primary care study. Am J Public Health.

[6] Santamarina-Perez P, Eiroa-Orosa FJ, Rodriguez-Urrutia A, Qureshi A, Alegre J (2014). Neuropsychological impairment in female patients with chronic fatigue syndrome: a preliminary study. Appl Neuropsychol Adult.

[7] Murga I, Aranburu L, Gargiulo PA, Gómez Esteban JC, Lafuente JV (2021). Clinical heterogeneity in ME/CFS. A way to understand long-COVID19 fatigue. Front Psychiatry..

[8] Teodoro T, Edwards MJ, Isaacs JD (2018). A unifying theory for cognitive abnormalities in functional neurological disorders, fibromyalgia and chronic fatigue syndrome: systematic review. J Neurol Neurosurg Psychiatry.

[9] Ocon AJ (2013). Caught in the thickness of brain fog: exploring the cognitive symptoms of Chronic Fatigue Syndrome. Front Physiol.

[10] Christley Y, Duffy T, Everall IP, Martin CR (2013). The neuropsychiatric and neuropsychological features of chronic fatigue syndrome: revisiting the enigma. Curr Psychiatry Rep.

[11] Fujii H, Sato W, Kimura Y (2020). Altered structural brain networks related to adrenergic/muscarinic receptor autoantibodies in chronic fatigue syndrome. J Neuroimaging.

[12] Glassford JAG (2017). The neuroinflammatory etiopathology of myalgic encephalomyelitis/chronic fatigue syndrome (ME/CFS). Front Physiol.

[13] Noda M, Ifuku M, Hossain MS, Katafuchi T (2018). Glial activation and expression of the serotonin transporter in chronic fatigue syndrome. Front Psych.

[14] Nater UM, Lin JMS, Maloney EM (2009). Psychiatric comorbidity in persons with chronic fatigue syndrome identified from the eorgia population. Psychosom Med.

[15] Santamarina-Pérez P, Freniche V, Eiroa-Orosa FJ (2011). El rol de la depresión en el déficit cognitivo del paciente con síndrome de fatiga crónica. Med Clin.

[16] Sivan M, Taylor S (2020). NICE guideline on long covid. BMJ.

[17] Shepherd C (2021). Long Covid and Me/Cfs.

[18] Soriano JB, Murthy S, Marshall JC, Relan P, Diaz J (2021). A clinical case definition of post-COVID-19 condition by a Delphi consensus. Lancet Infect Dis.

[19] Zayet S, Zahra H, Royer PY (2021). Post-COVID-19 syndrome: nine months after SARS-CoV-2 infection in a cohort of 354 patients: data from the first wave of COVID-19 in nord franche-comté hospital, France. Microorganisms..

[20] Islam MF, Cotler J, Jason LA (2020). Post-viral fatigue and COVID-19: lessons from past epidemics. Fatigue Biomed Health Behav.

[21] Fernández-de-las-Peñas C, Palacios-Ceña D, Gómez-Mayordomo V (2021). Prevalence of post-COVID-19 symptoms in hospitalized and non-hospitalized COVID-19 survivors: a systematic review and meta-analysis. Eur J Intern Med.

[22] Montenegro P, Moral I, Puy A (2022). Prevalence of post COVID-19 condition in primary care : a cross sectional study. Int J Environ Res Public Health.

[23] Antonelli M, Penfold RS, Merino J (2022). Risk factors and disease profile of post-vaccination SARS-CoV-2 infection in UK users of the COVID Symptom Study app: a prospective, community-based, nested, case-control study. Lancet Infect Dis.

[24] Komaroff AL, Lipkin WI (2021). Insights from myalgic encephalomyelitis/chronic fatigue syndrome may help unravel the pathogenesis of postacute COVID-19 syndrome. Trends Mol Med.

[25] Mantovani E, Mariotto S, Gabbiani D (2021). Chronic fatigue syndrome: an emerging sequela in COVID-19 survivors?. J Neurovirol.

[26] Tanaka M, Mizuno K, Yamaguti K (2011). Autonomic nervous alterations associated with daily level of fatigue. Behav Brain Funct.

[27] Słomko J, Estévez-López F, Kujawski S (2020). Autonomic phenotypes in chronic fatigue syndrome (Cfs) are associated with illness severity: a cluster analysis. J Clin Med.

[28] Moore RD, Romine MW, O’Connor PJ, Tomporowski PD (2012). The influence of exercise-induced fatigue on cognitive function. J Sports Sci.

[29] Murga I, Aranburu L, Gargiulo PA, Gómez-Esteban JC, Lafuente JV (2021). The maintained attention assessment in patients affected by Myalgic encephalomyelitis/chronic fatigue syndrome: a reliable biomarker?. J Transl Med.

[30] Dick BD, Rashiq S (2007). Disruption of attention and working memory traces in individuals with chronic pain. Anesth Analg.

[31] Hart RP, Martelli MF, Zasler ND (2000). Chronic pain and neuropsychological functioning. Neuropsychol Rev.

[32] Rodriguez-Raecke R, Niemeier A, Ihle K, Ruether W, May A (2009). Brain gray matter decrease in chronic pain is the consequence and not the cause of pain. J Neurosci.

[33] Zamponi HP, Juarez-Aguaysol L, Kukoc G (2021). Olfactory dysfunction and chronic cognitive impairment following SARS-CoV-2 infection in a sample of older adults from the Andes mountains of Argentina. Alzheimer’s & Dementia.

[34] Pirker-Kees A, Platho-Elwischger K, Hafner S, Redlich K, Baumgartner C (2021). Hyposmia is associated with reduced cognitive function in COVID-19: first preliminary results. Dement Geriatr Cogn Disord.

[35] Douaud G, Lee S, Alfaro-Almagro F (2022). SARS-CoV-2 is associated with changes in brain structure in UK Biobank. Nature.

[36] Wilckens KA, Woo SG, Kirk AR, Erickson KI, Wheeler ME. Role of sleep continuity and total sleep time in executive function across the adult lifespan. Psychol Aging. 2014;29(3):658–65. 10.1037/a0037234. 10.1037/a0037234 PMC4369772 25244484

[37] Yaffe K, Falvey CM, Hoang T (2014). Connections between sleep and cognition in older adults. Lancet Neurol.

[38] Hsieh ST, Anand P, Gibbons CH, Sommer C, Anand P, Gibbons CH, Sommer C, Hsieh ST (2019). Small fiber neuropathy and related syndromes: pain and neurodegeneration. Small fiber neuropathy and related syndromes: pain and neurodegeneration.

[39] Kraychete DC, Sakata RK (2011). Neuropatias Periféricas Dolorosas. Rev Bras Anestesiol.

[40] Blitshteyn S, Whitelaw S (2021). Postural orthostatic tachycardia syndrome (POTS) and other autonomic disorders after COVID-19 infection: a case series of 20 patients. Immunol Res.

[41] Chu L, Valencia IJ, Garvert DW, Montoya JG (2019). Onset patterns and course of myalgic encephalomyelitis/chronic fatigue syndrome. Front Pediatr.

[42] Devendorf AR, McManimen SL, Jason LA (2020). Suicidal ideation in non-depressed individuals: the effects of a chronic, misunderstood illness. J Health Psychol.

[43] Chu L, Elliott M, Stein E, Jason LA (2021). Identifying and managing suicidality in myalgic encephalomyelitis/chronic fatigue syndrome. Healthcare (Switzerland).

[44] McManimen SL, McClellan D, Stoothoff J, Jason LA (2018). Effects of unsupportive social interactions, stigma, and symptoms on patients with myalgic encephalomyelitis and chronic fatigue syndrome. J Commun Psychol.

